# Introduction to a Landscape Analysis of Multisectoral Approaches for Prevention and Control of Infectious and Vector-Borne Diseases

**DOI:** 10.1093/infdis/jiaa489

**Published:** 2020-10-29

**Authors:** Florence Fouque, Karin Gross, Zee Leung, Konstantina Boutsika

**Affiliations:** 1 UNICEF/UNDP/World Bank/WHO Special Programme for Research and Training in Tropical Diseases (TDR), World Health Organization, Geneva, Switzerland; 2 Swiss Agency for Development and Cooperation, Switzerland; 3 International Development Research Centre, Canada; 4 Swiss Tropical and Public Health Institute, Basel, Switzerland; 5 University of Basel, Basel, Switzerland

**Keywords:** vector-borne diseases, prevention, control, multisectoral approaches

## Abstract

The Swiss Development Cooperation, Canada’s International Development Research Centre, the Swiss Tropical Public Health Institute, and the UNICEF/United Nations Development Programme (UNDP)/World Bank/World Health Organization (WHO) Special Programme for Research and Training in Tropical Diseases (TDR) collaborated on a project to review, understand and promote the use of multisectoral approaches (MSAs) in the prevention and control of vector-borne diseases (VBDs). The objectives of the project were to support a landscape analysis of how MSAs have been used in the prevention and control of VBDs; to develop a theoretical framework for guiding the implementation of interventions; and to test the recommendations in real-life conditions. To realize these objectives, the project supported several activities, including commissioning a series of scientific reviews on MSAs in 5 thematic areas, sharing the key findings of these reviews in workshops and events, and developing a guidance framework for the implementation of MSAs. These activities have produced the theoretical framework that will be tested in real-life conditions through the support of case studies. The collaboration on implementing multisectoral activities against VBDs will continue among TDR, the Swiss Tropical Public Health Institute, and new partners such as the WHO Water Sanitation and Hygiene Group, UNDP, and UN-Habitat, in order to face the challenges identified and propose solutions tailored to specific contexts. The prevention and control of VBDs require strong and adapted MSAs with the full participation of all relevant sectors.

The burden of infectious diseases has drastically decreased in the past 50 years, but it still represents the major cause of premature death in the world and is expected to remain so until 2030, with 41 million deaths annually [1]. Vector-borne diseases (VBDs), including malaria and emerging arboviral diseases, account for about one-quarter of all infectious diseases [2], and the significant progress against malaria are halting since a few years. Further, the rapid expansion of other diseases. including those caused by arboviruses such as dengue, chikungunya, yellow fever, and Zika, is shown by exponential increases in numbers of cases and fatalities.

The emergence, transmission, and distribution of VBDs are linked to a wide range of intertwined and partially overlapping factors that belong to multiple sectors—from the biological elements of the system, such as pathogen and vector characteristics, to social and global elements, such as poverty, human behavior, and climate change. It has become evident that the prevention and control of these diseases must be driven by more than a single approach, because transmission patterns are driven by vector-host-pathogen relationships in which natural conditions, human societies and vector parameters are dynamically interacting and changing. In this context, a multisectoral approach (MSA) is required to effectively address these complex and dynamic transmission patterns.

A first framework for MSA against malaria was developed by the Roll Back Malaria (RBM Partnership) in collaboration with United Nations Development Program (UNDP) through the Multisectoral Action Framework for Malaria (MAFM), published in 2013 and revised in 2019 [3]. The MAFM calls for action at several levels and in multiple sectors, globally and across international and intranational boundaries, and by different organizations. It emphasizes complementarity, effectiveness, and sustainability, and it involves new interventions as well as putting new life into those that already exist, coordinating and managing these in new and innovative ways. With the purpose to expand the MAFM to other VBDs, the Swiss Development Cooperation (SDC) and the Swiss Tropical Public Health Institute (Swiss TPH) developed a concept note on which the current project was based.

The SDC, the International Development Research Centre (IDRC) from Canada, the Swiss TPH, and the UNICEF/United Nations Development Programme (UNDP)/World Bank/World Health Organization (WHO) Special Programme for Research and Training in Tropical Diseases (TDR) agreed on a collaborative activity, started in late 2016, to better understand the landscape, the building blocks, and the processes of an MSA for the prevention and control of VBDs and to implement selected case studies to test these approaches. The objectives of this project were (1) to synthesize global knowledge on current multisectoral activities that are deployed in different regions against infectious diseases, (2) to draw theoretical processes and general recommendations from experiences, and (3) to test the recommendations into real-life conditions through case studies.

The project was then structured to include different steps (Figure 1). The first step was the support of commissioned reviews on specific related topics; the second was the organization of joint meetings, workshops, and events to discuss and put together all relevant sectors; and the third was the development of a guidance framework for implementing of MSAs. These first 3 steps have been fully completed in the past 3 years and were used to draw the final step of the project. This last step is currently ongoing and will follow the implementation of case studies on MSAs against different diseases in low- and middle-incomes countries to test the theoretical framework.

**Figure 1.**
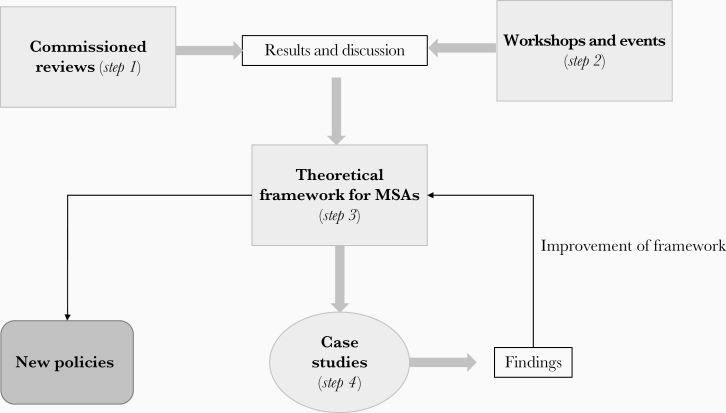
Steps of the project on multisectoral approaches (MSAs) for prevention and control of vector-borne diseases, with steps 1–3 already achieved and step 4 planned for the coming years.

## ACTIVITIES

### Commissioned Reviews

A call was launched in January 2017 to support 6 commissioned reviews on specific topics related to MSAs for the prevention and control of VBDs. The overall objective of the call was to support a landscape analysis on examples of MSAs to prevent and control VBD transmission and to identify knowledge gaps. The commissioned reviews were mandated to investigate current knowledge and experiences on topics related to different sectors. The publications on some of the main findings from the reviews are included in the current supplement to *The Journal of Infectious Diseases,* and the abstracts from the final technical reports are provided below.

***Review 1: Impact of the industrial sector on VBDs, with the example of gold mining activities disrupting malaria ecosystems in Africa and the Americas.*** The objective of this review was to retrieve and analyze available data and information on the impact of industrial activities on VBDs transmission, with a special focus on mining activities that can greatly disturb existing malaria ecosystems in Africa, Asia and Latin America. The information presented in this review has already been made available to improve policies, practices, and research priorities going forward [4]. Some key recommendations from the findings include establishing a national interministerial task force for vector control, promoting the building of suitable and location-specific multisectoral partnerships, mandating the use of health impact assessments and/or expanding the environmental impact assessments to health requirements, reaching the informal sector through local health posts and roaming healthcare workers, conducting risk mapping to determine transmission risks, ensuring community engagement and social outreach when implementing vector control programs, making health messaging and education a component of disease control efforts, and monitoring and evaluating VBD programs.

***Review 2: Dengue virus as a proxy to describe and assess the individual and combined impact of vector control strategies, including within the ecobiosocial context.*** Weighted and prioritized integrated strategies for the prevention and control of VBDs within the context of ecobiosocial approaches provided evidence for progressive implementation of more comprehensive ways to control VBDs. High-level commitment of multiple ministries is central to the intersectoral interactions required to plan, fund, and implement prioritized activities outlined in the response. Sustained political engagement will be required to maintain momentum for systems reforms required for adjustment to an integrated approach. Prospective primary field studies are needed to generate evidence addressing the impact of integrated strategies to prevent and control VBDs within the context of ecobiosocial approaches and the multisectoral participation. In addition, the usefulness of MSA was found to go beyond current public health VBD threats to emerging and reemerging ones, especially viral diseases.

***Review 3: Impact of human mobility (individual or population) caused by economic, civil unrest, or war reasons, displacement of temporary workers, and other population movements, on the emergence of arboviral outbreaks***. Population displacement and other forced movement patterns after natural disasters or armed conflicts or due to socioeconomic reasons were found to contribute significantly to the global emergence of *Aedes*-borne viral disease epidemics. Dengue and chikungunya epidemiology are critically affected by situations of displacement and forced movement patterns, within and across borders. In this respect, waves of human movements have been a major driver for the changing epidemiology and outbreaks of the disease on local, regional, and global scales. Both emerging dengue autochthonous transmission and outbreaks in countries known to be nonendemic and cocirculation and hyperendemicity with multiple dengue virus serotypes have led to the emergence of severe disease forms, such as dengue hemorrhagic fever and dengue shock syndrome. The same was also found with atypical and severe forms of chikungunya when emerging in outbreaks due to human mobility and affecting nonimmune populations in new territories.

***Review 4: Role of the different stakeholders in a multisectoral intervention to distribute insecticide-treated nets to mobile populations in Southeast Asia***. The review was focusing on the vulnerable groups, such as the mobile and migrant population (MMP) in Myanmar, which are now the focal malaria transmission groups, impeding malaria elimination in the country. To control malaria transmission and achieve subsequent malaria elimination, one of the interventions focused on increasing use of personal protective measures, such as insecticide-treated bed nets (ITNs) for the MMPs in Myanmar artemisinin resistance containment zones. The objectives of this study were to (1) identify which stakeholders were involved in intersectoral approaches to support the intervention of increasing access to and use of ITNs targeted at the MMPs in these zones, (2) characterize the ITN interventions targeted to these special groups of the population, and (3) analyze how the intersectoral collaboration was deployed and how this approach was supported in the target population.

The findings show that interventions to distribute ITNs for the prevention of malaria were supported by multiple stakeholders (local, national, nongovernmental organizations, and others); however, it was not described how the intersectoral collaboration was working. Nevertheless, the net ownership and rates of use at the end of the project did not met the WHO targets for Myanmar. Further data were missing to specifically assess the role of the different stakeholders involved in the interventions. This review clearly demonstrates some gaps in looking at multisectoral collaboration, as well as the absence of specific indicators to show how MSA was working and whether the failure was due to faulty implementation of the approach or to other factors.

***Review 5: Examination of how stakeholders work together to implement a global multisectoral strategy.*** Although WHO is recommending intersectoral collaboration as one of the key elements of integrated vector management and assumed this would make an important contribution to VBDs control and elimination, there is limited evidence comparing the effect and contribution of intersectoral approaches with those of the health sector only. For that purpose, a systematic review from more than 40 years of scientific literature was undertaken to develop an evidence-based framework of intersectoral collaboration and assess its effectiveness in sustaining the prevention and control of VBDs.

Among the 50 articles included in the analysis, 19 were categorized as of moderately strong quality. All articles compared preintervention and postintervention outcomes against disease or vector variables (with the intervention being intersectoral collaboration). Three articles included outcome variables on intersectoral collaboration and participation indicators. However, analysis by different sectors or different activities was retrieved. Only 1 article compared cost data for community-intersectoral intervention for Indoors Residual Spraying (IRS) and traditional “vertical” IRS. Six factors extracted from 47 studies were identified as influencing the effectiveness of intersectoral collaboration. The main ones were using approach factor (in 37 studies out of 47), resources (in 34 studies out of 47), relationships (in 33 studies out of 47), and management (in 29 studies out of of 47). The recent global strategy of VBD control and prevention encourages intersectoral collaboration as an approach to achieving cost-effective and efficient results from an intervention. The review showed that although intersectoral collaboration has played an important role in achieving reduction of VBDs or vector densities, very few studies have measured how much intersectoral collaboration contributed to this impact, and the relevant indicators are missing [5].

***Review 6: Scoping review of intersectoral collaborations to prevent and control VBDs.*** This review synthesizes evidence for models of intersectoral collaborations for the prevention and control of VBDs. Half of the articles were about malaria control in the African region. The other half of the publications retrieved were on the prevention and control of dengue, with interventions based in Asia and Latin America. Among the many gaps or challenges that impeded successful implementation and lowered the chances of project sustainability, most notably, the disconnect between different stakeholder responsibilities was often encountered and exemplified. The observation of a lack of communication between multilateral organizations and local governments, for whatever reason, is of great concern with a top-down approach that makes stakeholder relationships more susceptible to disconnection from field reality.

The ownership of the programs by the community was an issue, as multiple cases cited the lack of understanding, interest, and initiative as a reason for discontinuity. This observation emphasizes the needs of MSAs, including local and community sectors. The lack of research capacity, including baseline data, skilled and knowledgeable staff, and models for data analysis that could be contextualized to local needs, was evident for both malaria and dengue control programs. The overall results show the need for a comprehensive framework for an effective and sustainable MSA to prevent and control VBDs. Because both intersectoral collaboration and VBDs are broad topics that hinge on social and economic development, the issues of financing, investment in human resource development, and supply of materials should be addressed through the collaboration. The Delphi-validated statements summarizing recommendations for MSAs are globally applicable, but they need to be contextualized to a national and even municipal level.

### Workshops and Events

***TDR-SDC-IDRC-Swiss TPH Workshop on Multisectoral Approaches for Prevention and Control of Vector-Borne Diseases: Current Knowledge and Research Gaps and Priorities, Geneva, Switzerland, 26–28 June 2017.*** The workshop was attended by about 40 participants and had a specific session with WHO member states. some activities were recognized by the participants as priority activities to be undertaken once the commissioned reviews have produced their results, through final technical reports and publications. These activities included specific work to support the MSA for prevention and control of VBDs and recommendations at different levels, including global, cross-border, national, and local.

The recommendations from the workshop included the establishment of a guiding and advocacy framework as well as support for case studies to collect evidence on MSA potential and functioning, including collection and sharing of data, analysis, cost estimates, overall health impacts, and risks for potential outbreaks. The recommendations for international agencies and national/local levels included a requirement for assessment of vector control needs; coordination of health impact assessment; and operationalization of the MSA at the country level, creating a coordinated system that is ready to respond to different VBD situations, including when displaced people are affected or spread VBDs.

***Geneva Health Forum 2018: Global Health Security—Towards Multisectoral Collaborations to Confront the Increasing Threat of Vector-Borne Diseases, Geneva, Switzerland, 11 April 2018***. The aims of the session were an introduction to the rationale of MSAs for VBDs; the presentation of key results from the commissioned reviews by the speakers; a discussion on successes, challenges, gaps, needs, and opportunities; and illustration of the potential beneficial impact of follow-up activities. The session began with a general introduction on the burden of VBDs, the available evidence that MSAs are needed to prevent and control VBDs needs MSAs, and the need to know where we are in conceptualizing and implementing these approaches. Rashad Abdul-Ghani (PhD) presented the review on chikungunya virus as a globally (re)emerging and rapidly expanding epidemic threat driven by human mobility patterns, showing that the expansion of this virus is strongly linked to human movement, which in turn is related to social, economic, political (multisectoral) factors. The warning that what is happening with chikungunya virus may also happen with any other virus, maybe more virulent, truly anticipated the current coronavirus disease 2019 pandemic.

Alfonso Rodriguez Morales (PhD) presented the integrated strategies for the prevention and control of VBDs within the context of ecobiosocial approaches, showing how to move from a single-oriented control of VBDs (vector control for example) to a multisectoral one. The single-oriented control take into account only one sector and one approach such health sector and vector control, opposite to a multisectoral one taking into consideration more than one sector such health and water and more than a single approach such as vector control and water management. The added value of integrating traditional vector control activities (health system) with newer technologies (community involvement, social approaches) was discussed, along with how these activities and approaches can be synergized and the associated challenges. Also discussed were recommendations on the way forward and potential opportunities for multisectoral research and action to help prevent and control VBDs. Jana Fitria Kartika Sari presented intersectoral collaboration for the prevention and control of VBDs, supporting the implementation of a global strategy, with some examples of success when working outside the health sector (eg, including education, agriculture, etc) and the remaining knowledge gaps. The final discussion introduced the global vector control response, which advocates for MSAs among the 4 pillars of the response [5].

***67th Annual Meeting of the American Society of Tropical Medicine & Hygiene: Poster presentation on “Multisectoral Approaches to Prevent and Control Malaria and Arboviral Diseases,” New Orleans, Louisiana, 31 October 2018***. The annual meetings of the American Society of Tropical Medicine & Hygiene attract thousands of participants, so this was an ideal forum to expose this work to a wider audience before the planned peer-reviewed publication. The poster summarized the progress and suggested the expected outcomes and the next steps.

### Developing a Guidance Framework for Implementing the MSAs

This document [6] was produced following one of the main recommendations from the reviews and exchanges with the stakeholders. The document starts with 2 introductory chapters on VBD basics and MSAs. The determinants of VBDs are described and grouped into the following categories: pathogen and vector related, environmental and agroecological, economic and social, and health system related. Together, these determinants go beyond the ministries of health and the health sector, concerning many other sectors and stakeholders. Existing prevention and control methods were laid out with challenges highlighted, among which was the weakness of the health sector alone. There are opportunities for better coordinated actions, such as potential synergy with the global momentum of the Sustainable Development Goals, multiple entry points for interventions across sectors and diseases, and empowerment through more research.

The historical background of MSA was traced to reveal this inevitable growing consensus. Examples of existing MSA case studies were extracted from the commissioned reviews and briefly discussed. Chapters 3 and 4 present the conceptual framework and its components. The conceptual framework is named BET—for base, energy, and technical elements—and includes 7 components: pillars, dimensions, levels, resources, sectors, domains and enablers. These components envelope the ingredients to include in a customized and tailored MSA. Chapter 5 outlines the coordination pathway from step 1 to step 6. Roles of nongovernment sectors and bodies are discussed with a focus on nongovernmental and international organizations, private sectors, and communities. Because financing and legislation are an indispensable part of the entire coordination pathway, funding mechanisms and the types of norms and policies needed during a multisectoral collaboration are included in this chapter. The guidance also emphasizes integration and synergy with the existing institutional structure for MSA within the country as well as with global multinational and multisectoral efforts, such as those under the Health and Environment Linkages Initiative, Sustainable Development Goal–related programs, and the One Health Initiative.

Chapter 6 covers sectoral guidance. A sectoral pathway is intended to assist government ministries to plan and initiate their work according to an MSA, from defining the vision to sectoral monitoring and evaluation, including important steps such as aligning MSA activities with the sector’s existing activities. A nonexhaustive list of key sectors is included, along with health: environment, water and sanitation, agriculture and aquaculture, energy, housing, education and research, finance, and legislature. The concluding chapter of the guidance document highlights the need for a system to monitor and evaluate these approaches and the interventions.

## DISCUSSION AND CONCLUSIONS

The activities developed around the analysis of MSAs for prevention and control of VBDs have resulted in new evidences retrieved from the analyses of the available publications and findings on this topic. Several thousands of articles were retrieved, screened, selected, and analyzed through the work done by the 6 commissioned reviews. The findings were published and some of them are included in this special issue. The main results were discussed over several events and exchanges with stakeholders from different sectors and levels (from global to local). Among the main challenges identified to building up an effective and efficient MSA, the lack of pathway and framework was considered critical, but the development of such a theoretical framework was feasible and was then achieved. This guidance document has now been published and needs to be tested and improved in real-life conditions.

Although the guidance is primarily directed to decision makers, with specific relevance for governmental sectors, the framework can be tailored to suit the needs of subnational and decentralized stakeholders. The purpose of this framework is not only to support supraministerial leaders and health sector but also to enhance the capacity of decision makers in other sectors to achieve in a collective effort efficient prevention and control of VBDs. Because the guidance document aims to delineate “how to,” apart from describing the essential components, recommendations are provided—for instance, on how to mobilize political will, how to enhance commitment and coordination among sectors, and how to strengthen community engagement. These elements will be studied in the following step of the project within specific cases of multisectoral collaboration to control specific diseases, such as malaria and arboviral diseases.

For that purpose, research proposals will be supported through a partnership with one of the most relevant sectors linked to health and VBDs, that is the water, sanitation, and hygiene (WASH) sector. A proposal was developed in partnership with the WHO WASH group to reduce WASH-related disease of poverty with a primary focus on VBDs, through the following activities: (1) refining and promoting research for impact on multisectoral action for health, (2) increasing the impact of joint convening of WASH and health sectors, and (3) supporting the strengthening of health systems to better address infectious diseases of poverty in general and VBDs in particular. The project will include 2 work packages: work package 1 on strengthening the prevention and control of diseases of poverty through multisectoral collaboration, based on the latest research findings and WHO WASH norms, and work package 2 on strengthening health systems to better address infectious diseases of poverty through improved WASH in healthcare facilities and enhanced capacity to manage WASH services and engage in good hygiene practices.

The partnership around the multisectoral activities will continue between TDR, the Swiss TPH, and new partners, such as UNDP and UN Habitat for building stronger recommendations and better addressing the numerous challenges identified. From past experiences and evidences, it has become clear that the prevention and control of VBDs cannot be achieved and sustained through single-sector intervention(s) and without the full commitment of not only the responsible entities but also the communities involved. What MSAs are targeting is exactly this full participation of all relevant sectors according to their own incentives and needs.

## Note

***Potential conflicts of interest.*** All authors: No reported conflicts. All authors have submitted the ICMJE Form for Disclosure of Potential Conflicts of Interest. Conflicts that the editors consider relevant to the content of the manuscript have been disclosed.

***Supplement sponsorship.*** This Supplement is sponsored by TDR: the Special Programme for Research and Training in Tropical Diseases co-sponsored by the United Nations Children’s Fund (UNICEF), the United Nations Development Programme (UNDP), the World Bank and the World Health Organization (WHO).
